# Reduced sampling rate Kalman filters for carrier phase and frequency offset tracking in 200 Gbps 16 QAM coherent communication system

**DOI:** 10.1038/s41598-020-80822-z

**Published:** 2021-01-21

**Authors:** Srishti Sharma, Pradeep Kumar Krishnamurthy

**Affiliations:** Center for Laser and Photonics, Indian Institute of Technology Kanpur, Kanpur, Uttar Pradesh India

**Keywords:** Optics and photonics, Optical techniques, Other photonics

## Abstract

We propose 1 state and 2 state multi-step Kalman filters (MKFs) to estimate and compensate CFO, LPN and NLPN in long-haul coherent fiber-optic communication systems. The proposed filters generate state estimates once every *m* symbols and therefore operate at a reduced sampling rate compared to conventional KFs that perform symbol by symbol processing. No computations are performed to obtain phase estimates of the intermediate $m-1$ samples; instead, the present and previous estimates are averaged and used to derotate the intermediate $m-1$ samples which are then demodulated to recover the transmitted symbols. This reduces the computational load on the receiver DSP. Further, in order to improve estimation accuracy, we adaptively vary the process noise covariance *Q*. Simulation results of 200 Gbps PDM 16 QAM system over 12 spans shows that the proposed 1 state MKF can reduce the sampling rate requirement by a factor of $m = 20$ with Q-factor degradation of 1.32 dB compared to single-step KF at linewidth of 100 kHz. The 2 state MKF tracks PN and CFO with a maximum step size of $m=10$ for a CFO of 100 MHz at linewidth of 100 kHz. We also study the dynamic performance of the proposed algorithms by applying step change to CFO. The 2 state MKF with adaptive *Q* is able to track a step change of 400 MHz of CFO with $m = 1$ and 3 with high estimation accuracy but slower convergence time compared to the non-adaptive 2 state MKF. Finally, we study the computational requirements of the proposed MKFs and show that they offer significant reduction in computations compared to single-step KF thus making the proposed filters suitable for hardware implementation.

## Introduction

Carrier synchronization is essential to demodulation of advanced modulation formats such as quadrature amplitude modulation (QAM) in high-data rate coherent optical communication links^1^. By carrier synchronization we mean that the effects of carrier frequency offset (CFO) between transmitter laser and receiver local oscillator (LO) laser and phase noise (PN) due to lasers (both transmit and LO) and nonlinear phase noise arising out of the interaction of amplified spontaneous emission (ASE) noise and Kerr nonlinearity in the fiber are estimated and compensated by the receiver. CFO and PN cause the received constellation to rotate from its original position thereby making it impossible to demodulate the received symbols correctly unless these effects are compensated^2^. CFO is estimated by techniques such as time-domain differential phase method and blind frequency search^3–5^. Estimation techniques such as Viterbi-Viterbi method, blind phase search, Barycenter algorithm, and QPSK partitioning are used to estimate carrier phase after adapting them to the higher-order QAM formats^6–8^. While these techniques maintain spectral efficiency of the transmission system as pilot symbols are not required for phase estimation, they are computationally expensive. Further, the phase estimation accuracy is limited by the quantization of test phases in BPS type algorithms which in turn limits the linewidth tolerance of the algorithm. The accuracy can be improved by employing additional stages and carrying out maximum likelihood phase estimation at the expense of increased computational complexity of the algorithm. Note that residual CFO affects the performance of the phase estimation algorithms. Once initial CFO and phase estimates are available, decision-directed feedback phase estimation can be advantageously employed for tracking residual CFO and phase errors^9,10^. It is possible to combine both blind and feedback based techniques into a two-stage carrier recovery algorithm in which the coarse CFO is estimated first using blind CFO estimator and the residual CFO and PN are estimated using a feedback based algorithm in the second stage. However, this introduces additional computational load on the receiver DSP.

It is also possible to perform joint estimation of CFO and PN instead of estimating them separately. Kalman filter (KF)^11^ and its variants such as extended^2,12^ and unscented Kalman filters^13^ have been demonstrated for joint estimation of CFO and PN. While they are in general superior over blind carrier recovery algorithms, they suffer from high computational complexity as they typically perform symbol by symbol state estimation. Moreover, symbol by symbol processing requires many computations per state estimation which introduces high latency in processing the symbols. This, combined with the sampling rate constraints of the CMOS ADCs^8,14^, makes it difficult to employ KFs in real-time processing. One method to overcome this problem is to employ block estimation techniques. Block KF^15^ and unscented KF algorithms^13^ have been proposed for joint estimation of CFO and carrier phase offset but do not perform well when laser and nonlinear phase noise is present in the received symbols. Moreover, these techniques are not studied for dynamic CFO estimation in which CFO changes suddenly to a new value during the transmission due to network issues. In this case, it is necessary that the carrier recovery algorithm converges to the correct value of CFO as quickly as possible which requires the study of tracking time of these algorithms. Finally, we note that the KF performance is sensitive to values of process noise covariance (*Q*) and measurement noise covariance (*R*). The estimation accuracy can be improved if *Q* and *R* can be estimated either before filter begins operation or adaptively during the filter operation^16^.

In this paper, we propose 1 state and 2 state multi-step Kalman filters (MKFs) for carrier recovery in 16 QAM 200 Gbps polarization division multiplexed coherent optical communication systems. In MKF, the state is updated once every *m* symbols in contrast to symbol by symbol state update of a conventional Kalman filter. The $m-1$ intermediate samples are discarded during phase estimation; instead they are derotated by the phase estimate obtained by averaging the current and previous MKF output. The derotated samples are then demodulated to recover the transmitted symbols. The 2 state MKF allows simultaneous estimation of CFO and phase noise which helps to lower the accuracy requirements of any CFO estimator that precedes MKF. We also show that the MKF algorithm requires only a few pilot symbols to track frequency and phase. In our simulations, only 20 pilot symbols were used in the training phase of the MKF algorithm. This does not affect the spectral efficiency of the transmission system as the training symbols is a negligible fraction of the total transmitted symbols. Further, we adaptively vary the process noise covariance *Q* to improve the estimation accuracy of the MKF. Updating the filter equations every *m* step significantly reduces the computational load on the receiver DSP and makes it possible to use KF techniques in practical implementation of carrier recovery for coherent communications.

The rest of the paper is organized as follows. In “Principle of 1 and 2 state multi-step Kalman filters” section, we describe the 1 and 2 state MKFs with adaptive Q. In “System model” section, we describe the simulation model of 200 Gbps 16 QAM coherent optical link. In “Results and discussion” section, we study performance of proposed MKFs in terms of maximum step size for given CFO and PN, maximum linewidth tolerance, and tracking time of dynamic CFO. We study dynamic CFO tracking performance of the proposed filters and show that the tracking time depends on the locations at which the CFO changes during transmission. We show that the filter converges rapidly when CFO change occurs during the initial transmission of symbols compared to the change occurring during the later part of the transmission. In “Computational complexity of MKFs” section, we compute the computational efficiency of MKF. Finally, in “Conclusion” section, we conclude by summarizing our results.

## Principle of 1 and 2 state multi-step Kalman filters

Carrier synchronization in coherent optical communications involves estimation and tracking of both carrier frequency offset and phase noise of the lasers. In addition, during transmission, the symbols are affected by nonlinear phase which consists of average phase shift due to self phase modulation (SPM) and stochastic phase noise due to the interaction of ASE noise of the amplifiers and the Kerr nonlinearity of the fiber.

The *k*th received sample after polarization mode dispersion (PMD) and chromatic dispersion (CD) compensation on ${\hat{x}}$ or ${\hat{y}}$ polarizations is given by

1 $$\begin{aligned} r_k=s_k e^{j\Psi _k}+n_k \end{aligned}$$

where $\Psi _k=\omega _k+\psi ^{PN}_k+\psi ^{SPM} _k +\psi ^{NLPN}_k$, $s_k$ is the transmitted complex symbol, $\omega _k=2\pi k\Delta f$ is the phase error due to CFO ($\Delta f$) of the local oscillator, $\psi ^{SPM} _k=2\gamma P_{in} L_{eff} N_s$ is the average phase due to SPM, $\psi ^{PN}_k$ is phase rotation due to PN, $\psi ^{NLPN}_k$ is phase due to NLPN, $\gamma$ is fiber nonlinear coefficient, $P_{in}$ is input launch power, $L_{eff}$ is effective length of the fiber and $n_k$ is ASE noise due to the inline optical amplifier which is modelled as zero-mean Gaussian random variable. In Eq. (1) we have modeled laser phase noise as discrete-time Wiener process with $\psi ^{PN}_k=\psi ^{PN}_{k-1} + \delta \psi ^{PN}_k$ in which $\delta \psi ^{PN}_k$ is the zero mean Gaussian random variable with variance $2 \pi \Delta \nu T_s$, where $\Delta \nu$ is the laser linewidth and $T_s$ is the symbol duration^17^.

In the following subsections we propose a 1 state MKF to track and estimate only laser phase noise and a 2 state MKF to jointly track and estimate PN and CFO.

### 1 state MKF

The state-space model of 1 state MKF to estimate phase $\Psi _k$ is given by the following equations.

2a $$\begin{aligned} \Psi ^p_k&=\Psi ^c_{k-m} +v_k \end{aligned}$$

2b $$\begin{aligned} Z_k&=\angle r_k s^*_k \end{aligned}$$

The state update equation is given by

3 $$\begin{aligned} \Psi ^c_k=\Psi ^c_{k-m}+K_k(Z_k-\Psi ^c_{k-m}) \end{aligned}$$

The equations for error covariance estimate *P* and Kalman gain *K* are given by

4a $$\begin{aligned} P^p_k&=P^c_{k-m}+Q \end{aligned}$$

4b $$\begin{aligned} P^c_k&=R{P^p}_k/(P^p_k+R) \end{aligned}$$

4c $$\begin{aligned} K_k&=P^p_k/(P^p_k+R) \end{aligned}$$

where *m* is the step size $(\ge 1)$, *P* is the error covariance, $v_k$ is zero mean Gaussian distributed noise of covariance *Q*, and *R* is the covariance of measurement noise. In the above equations superscript ‘*p*’ indicates predicted value and ‘*c*’ indicates corrected value. To initialise phase estimation, MKF is first operated in the data aided mode and then switched to the decision directed mode.

**Figure 1:**
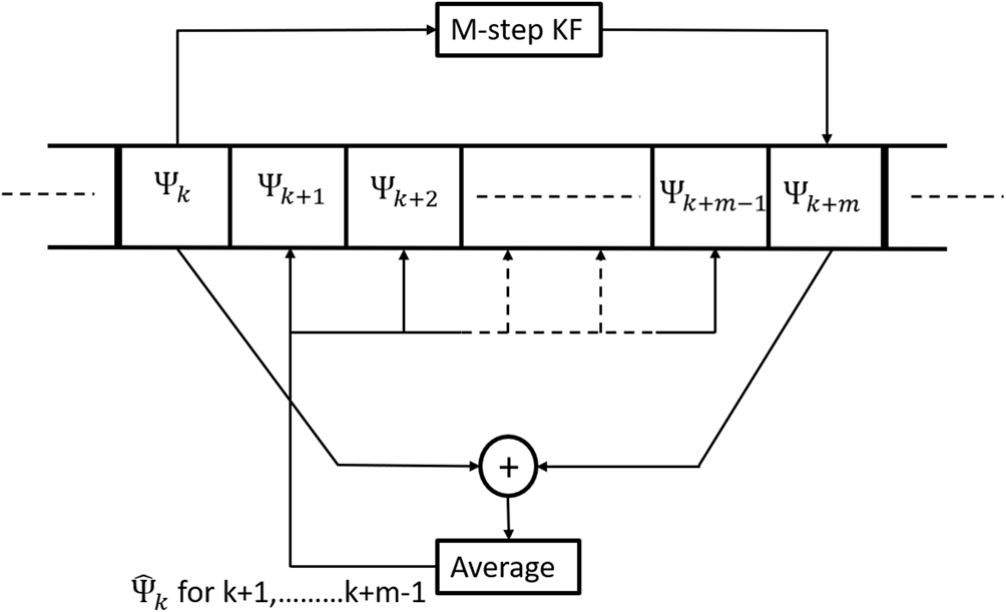
Principle of multi-step Kalman filter algorithm. Here *m* denotes the step size of the filter.

Figure 1 shows operation of the MKF algorithm with step-size *m*. The phase $\Psi _k^c$ is used to estimate the phase $\Psi _{k+m}^c$ using Kalman filter equations. The samples $r_{k+1}$, $r_{k+2}$,..., $r_{k+m-1}$ are derotated by the average of the phases $\Psi _k^c$ and $\Psi _{k+m}^c$ as shown in Fig. 1.

### 2 state MKF

The state space model for 2 state MKF is given by

5 $$\begin{aligned} \left(\begin{matrix} {\Psi }_{k}\\ {\omega }_{k} \end{matrix}\right) = \left(\begin{matrix} 1 &{} m\\ 0 &{} 1 \end{matrix}\right) \left(\begin{matrix} {\Psi }_{k-m}\\ {\omega }_{k-m} \end{matrix}\right) +\left( \begin{matrix} V_{1k}\\ V_{2k} \end{matrix}\right) \end{aligned}$$

Here $\Psi _k$ is the total phase error due to both PN and CFO, $\omega _k$ phase error due to CFO, $V_{1k}$ and $V_{2k}$ are the process noise which are Gaussian distributed. The following recursive MKF equations are used to obtain estimates of $\Psi _k$.

6a $$\begin{aligned} \Psi ^p_k&=\Psi ^c_{k-n}+ m\omega ^c_{k-n}+V_{1k} \end{aligned}$$

6b $$\begin{aligned} \omega ^p_k&=\omega ^c_{k-n}+ V_{2k} \end{aligned}$$

6c $$\begin{aligned} P^p_k&=AP^c_k A^T + Q \end{aligned}$$

6d $$\begin{aligned} K_k&=P^p_k H^T (HP^p H^T + R)^{-1} \end{aligned}$$

6e $$\begin{aligned} P^c_k&=P^p_k-K_kHP^p_k \end{aligned}$$

6f $$\begin{aligned} \delta \Psi&=tan^{-1}(Im(s_{1k}s_{2k}^*)/Re(s_{1k}s_{2k}^*)) \end{aligned}$$

6g $$\begin{aligned} \delta \omega&=Im(ks_{1k}s_{2k}^*)/Re(k^2 s_{1k}s_{2k}^*) \end{aligned}$$

6h $$\begin{aligned} \left(\begin{matrix} {\Psi }^c_{k}\\ {\omega }^c_{k} \end{matrix}\right) = & {} A \left(\begin{matrix} {\Psi }^c_{k-m}\\ {\omega }^c_{k-m} \end{matrix}\right) + K_k \left(\begin{matrix} \delta \Psi \\ \delta \omega \end{matrix}\right) \end{aligned}$$

where $K_k$ is the Kalman gain, *A* is state $2\times 2$ transition matrix given by $[1  m; 0 1]^T$ and $P_k$ is error covariance matrix. Just like 1 state MKF, in the above equations also, the superscript ‘*c*’ and ‘*p*’ indicates corrected value and predicted value of the concerned variable. *Q* and *R* are the covariance matrix of process and measurement noise respectively. $s_{1k}=r_k e^{(-j\Psi ^p)}$ and $s_{2k}$ is obtained from $s_{1k}$ after decision. $\delta \Psi$ and $\delta \omega$ are the residual for the total phase error and the phase error due to CFO. In the non-adaptive versions of 1 state and 2 state MKFs, we keep the covariance matrices *Q* and *R* constant throughout the filter operation.

### Adaptation of *Q*

In MKF, tracking capability and accuracy of the filter is sensitive to the value of the *Q* matrix. A poor choice of *Q* causes the filter to diverge thereby degrading its performance. In^16^ authors proposed an innovations based approach to adaptively change *Q* during filter operation in order to increase accuracy of state estimation. The innovation vector is given by $\delta r_k=r_k-{\hat{r}}_k$, where ${\hat{r}}_k$ is the $k^{th}$ predicted symbol. In this paper we apply this approach to our 2 state MKF. ${\hat{Q}}_k$, the innovation based approach estimate of *Q* at *k*th symbol time can be calculated as given in Eq. (7).

7 $$\begin{aligned}&{\hat{Q}}_k=E[{\hat{n}}_{k-m}{{\hat{n}}_{k-m}^T}]=KE[ \delta r_k{ \delta r_k}^T]K^T \end{aligned}$$

where *E*[.] is the expectation operator. To solve Eq. (7), the expectation operator is approximated by the time average value of $\delta r_k{ \delta r_k}^T$. In^18^, a forgetting factor $\beta$ is proposed to obtain the average value of $\delta r_k{ \delta r_k}^T$ over time. Therefore *Q* in Eq. (6c) can be adapted with the symbol time giving $Q_k$.

8 $$\begin{aligned} Q_k=\beta Q_{k-1} + (1-\beta ){\hat{Q}}_k \end{aligned}$$

The forgetting factor controls the performance of the filter in terms of convergence speed and estimation accuracy.

## System model

**Figure 2:**
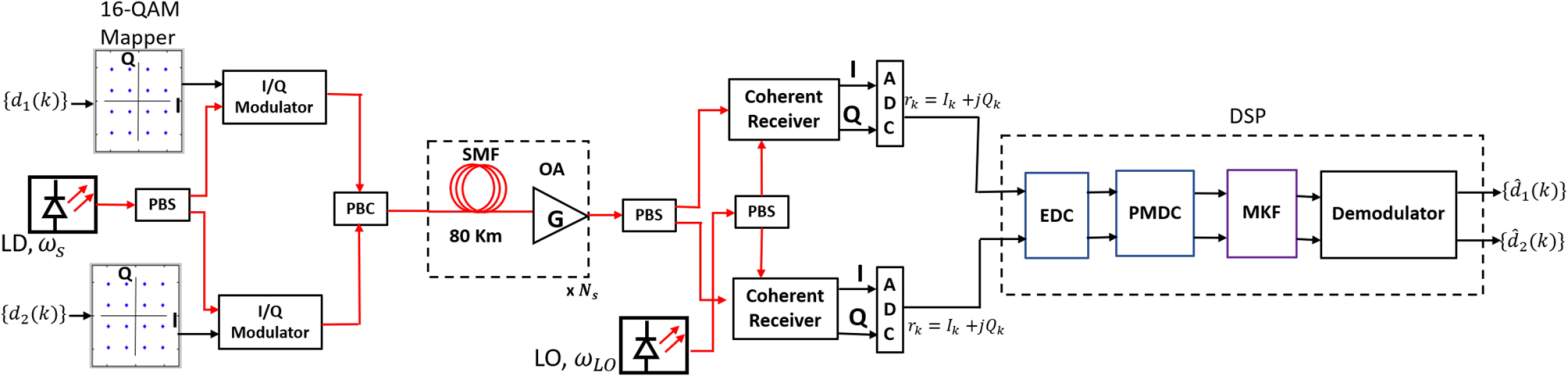
Simulation setup for 200 Gbps 16 QAM coherent optical communication system. SMF, single mode fiber; OA, optical amplifier; ADC, analog to digital convertor; EDC, electronic dispersion compensation; PMDC, polarisation mode dispersion compensation. PBS and PBC, polarisation beam splitter and combiner; MKF, multi-step Kalman filter.

In this section we describe the simulation setup of 200 Gbps polarization division multiplexed (PDM) 16 QAM single-channel coherent communication system as shown in Fig. 2 to characterize the performance of the filters described in “Principle of 1 and 2 state multi-step Kalman filters” section. At the transmitter, two symbol sequences $d_1(k)$ and $d_2(k)$ of length 10,000 each are generated and are mapped onto a square 16 QAM constellation. These complex symbols are then passed to the rectangular pulse shaper. These rectangular pulses in electrical domain are then modulated onto the ${\hat{x}}$ and ${\hat{y}}$ polarizations of optical carrier at 1550 nm using an optical I/Q modulator. A polarization beam combiner (PBC) is used to combine the modulated symbols of ${\hat{x}}$ and ${\hat{y}}$ polarizations to form the complex data stream to be transmitted. The complex symbols are then transmitted over an optical fiber link consisting of $N_s=12$ spans with each span comprising SSMF and inline optical amplifier to compensate for span losses. We model the propagation of the symbols by coupled nonlinear Schrodinger equation (CNLSE) which includes CD, PMD and SPM effects. In each span, fiber is divided into small sections and in each section CNLSE is numerically solved using split step Fourier method (SSFM). In order to model the effects of PMD, we consider each section of the fiber as a waveplate and using Jones matrix formalism we write the transmission matrix of the $i^{th}$ waveplate as given in Eq. (9).

9a $$M(-\theta _i,-\phi _i)\left( \begin{matrix} e^{-j\frac{\Psi }{2}} &{} 0\\ 0 &{} e^{j\frac{\Psi }{2}} \end{matrix}\right) M(\theta _i,\phi _i) $$

9b $$M(\theta _i,\phi _i)=\left( \begin{matrix} {\cos} \theta &{} {\sin} \theta e^{j\phi }\\ - {\sin}\theta e^{-j\phi } &{} {\cos}\theta \end{matrix}\right)$$

where $\Psi =\omega \Delta \tau _i$ in which $\Delta \tau _i$ is differential group delay (DGD) of $i^{th}$ waveplate and $\omega$ is the angular frequency. $\phi$ and $\theta$ are the elevation and azimuthal angle on the Poincare sphere^19^.

Each span consists of 80 km of SSMF giving a total of 960 km propagation. The SSMF parameters are: loss coefficient $\alpha =0.2$ dB/km, dispersion coefficient $D_s=17$ ps/nm-km, Kerr nonlinear coefficient $\gamma = 1.3$/W/km and PMD coefficient $D_p=0.1$ ps/$\sqrt{\text {km}}$. Each span of fiber is divided into 40 waveplates which are simulated using an open source software Optilux^24^ and transmission of symbols through waveplates is governed by Eq. (9). An inline optical amplifier of gain and noise figure of 16 dB and 5 dB respectively compensates for the span losses and adds ASE noise power within 0.1 nm reference bandwidth. We set $\beta =0.88$.

At the receiver, ${\hat{x}}$ and ${\hat{y}}$ polarizations are separated using PBS which are then coherently demodulated using the local oscillator and coherent receiver. PMD and CD are jointly compensated using the vector form of digital back propagation (DBP). The resulting complex symbols are passed to MKF for further processing. Performance is analysed in terms of Q-factor, calculated using EVM method given by the following equations^20,21^.

10a $$\begin{aligned} \text {BER}&={\frac{3}{8}}\text {erfc}{\sqrt{\sqrt{2}/10{\text {(EVM)}}^2}} \end{aligned}$$

10b $$\begin{aligned} \text {Q(dB)}&=20{\text {log}_{10}}(\sqrt{2}\text {erfc}^{-1}(2\text {BER})) \end{aligned}$$

## Results and discussion

**Figure 3:**
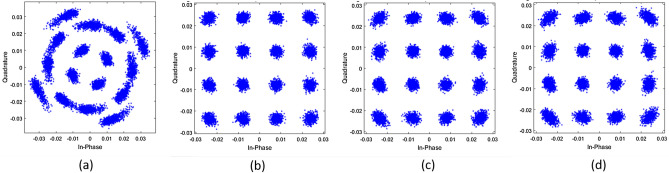
Constellation diagrams of the received signal on ${\hat{x}}$ -polarisation before and after 1 state MKF at $P_{in} = 1$ dBm, $N_s = 12$ spans, $\delta \nu = 100$ kHz. (**a**) PDM-16-QAM before 1 state MKF, (**b**) PDM-16-QAM after 1 state MKF for $m=1$, (**c**) $m=10$, (**d**) $m=20$.

**Figure 4:**
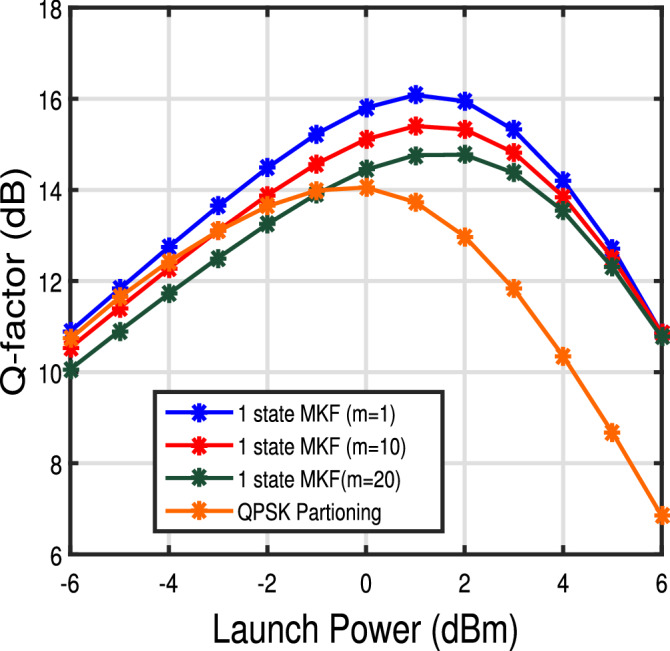
Q-factor versus launch power curve for 1 state MKF for $\Delta \nu = 100$ kHz and 12 spans for different step sizes.

**Figure 5:**
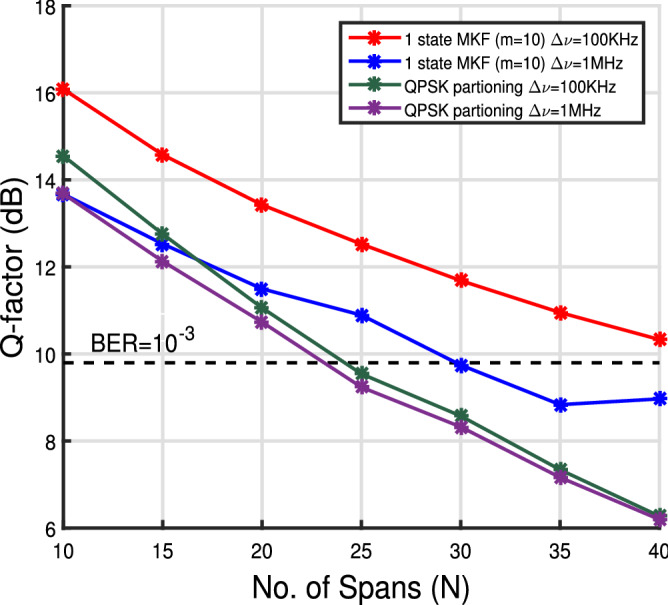
Q-factor versus no. of spans curve for 1 state MKF for $\Delta \nu = 100$ kHz and launch power of 1 dBm. For reference, Q-factor corresponding to the BER of $10^{-3}$ is shown.

**Figure 6:**
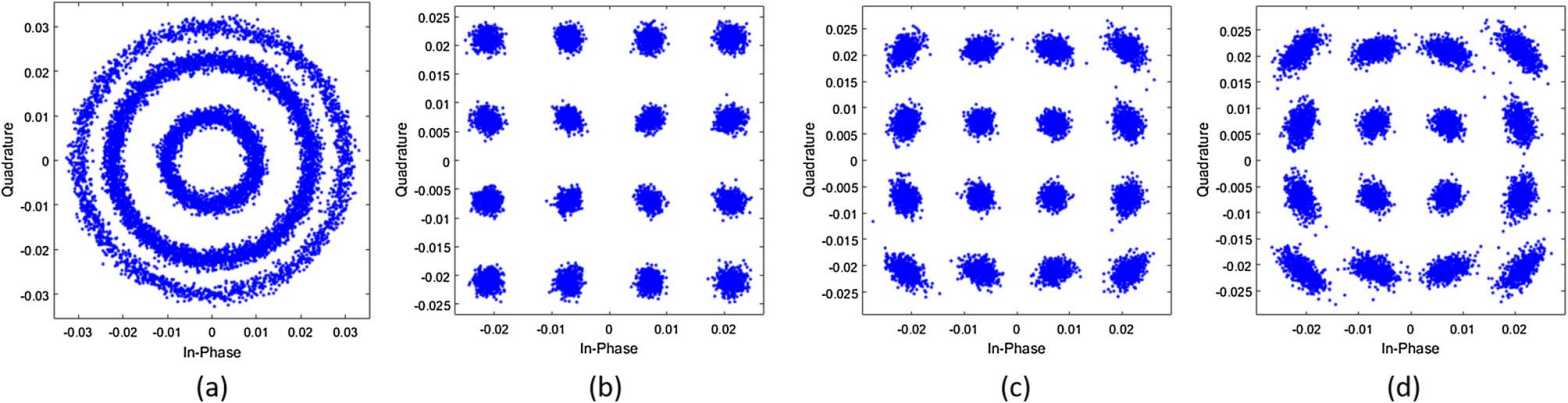
Constellation diagrams of the received signal on ${\hat{x}}$-polarisation before and after 2 state MKF at Pin = 0 dBm, $N_s = 12$ spans, $\Delta \nu = 100$ kHz. (**a**) PDM-16-QAM before 2 state MKF, (**b**) PDM-16-QAM after 2 state MKF for $m=1$ (**c**) $m=5$, (**d**) $m=10$.

**Figure 7:**
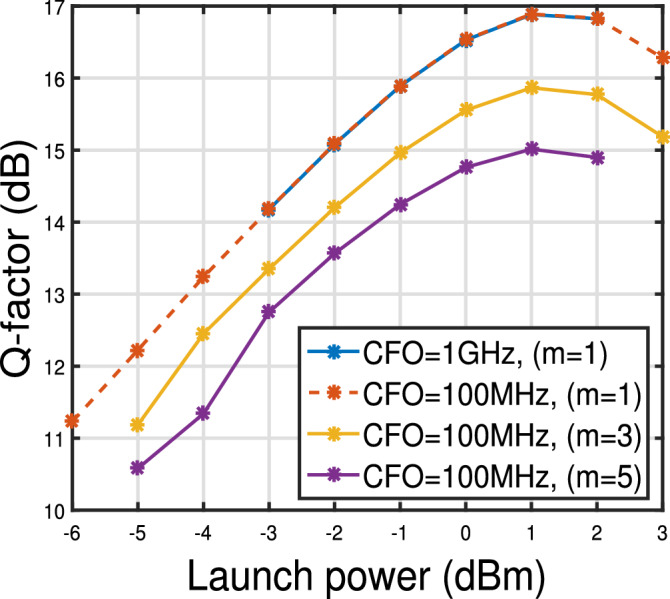
Q-factor versus launch power curve for 2 state MKF for $\Delta \nu = 100$ kHz and 12 spans for different CFO values and step sizes (*m*).

**Figure 8:**
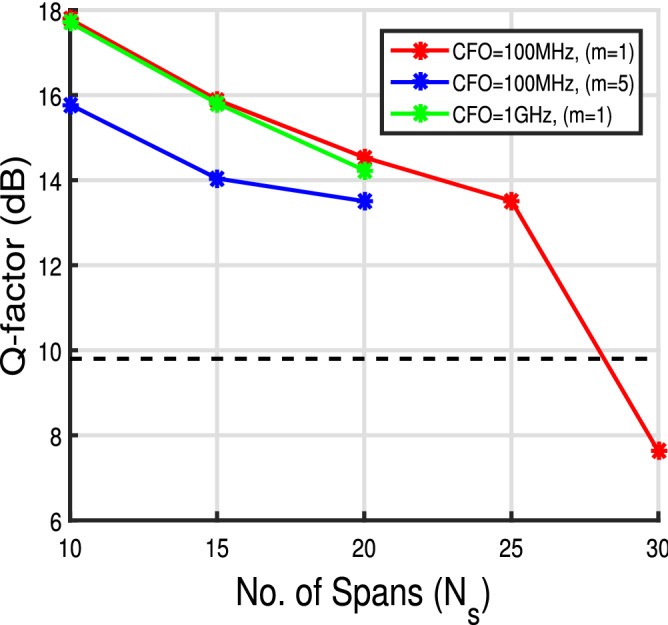
Q-factor versus no. of spans curve for 2 state MKF for $\Delta \nu = 100$ kHz and launch power of 1 dBm.

**Figure 9:**
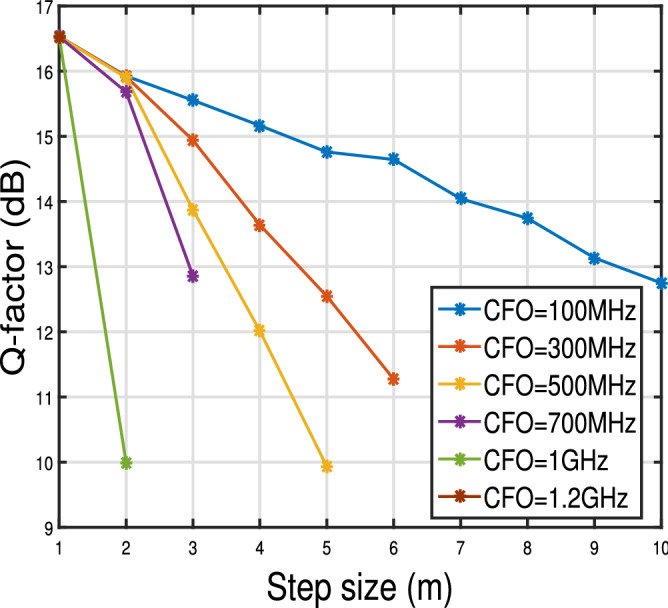
Q-factor versus step size of 2 state MKF for $\Delta \nu = 100$ kHz and different frequency offsets for Pin = 0 dBm. For reference, Q-factor corresponding to $10^{-3}$ BER is shown.

**Figure 10:**
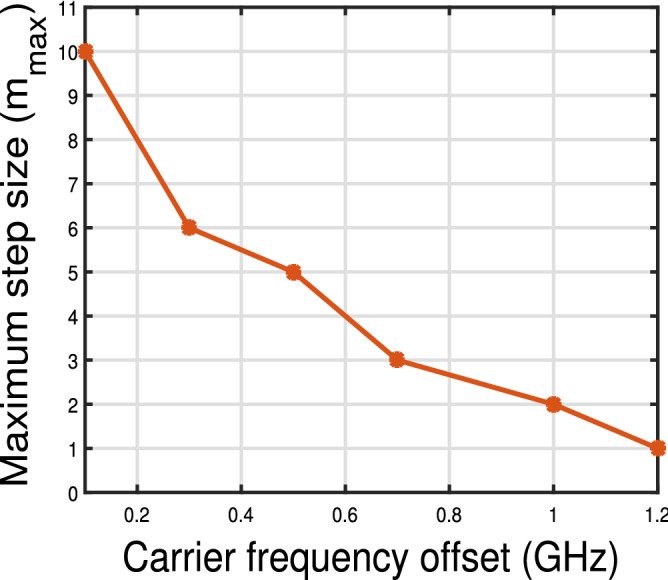
Maximum achievable step size for various frequency offsets for 2 state MKF.

**Figure 11:**
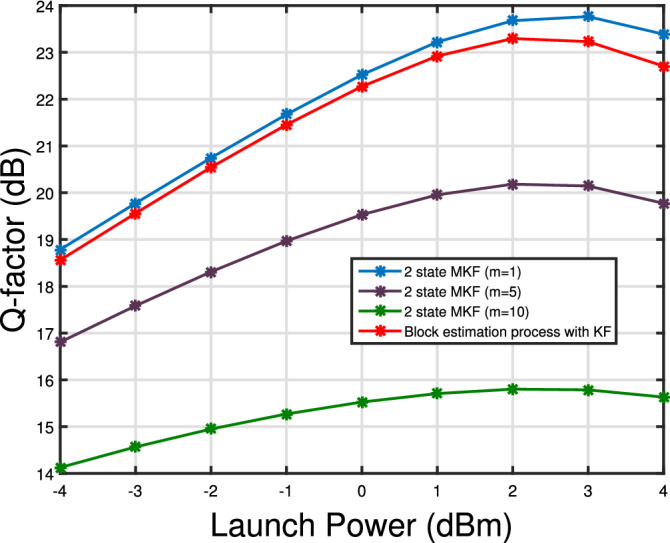
Q-factor versus launch power curve for 2 state MKF for CFO = 100 MHz and 3 spans. For 2 state MKF $\Delta \nu = 100$ kHz, For block estimation of KF $\Delta \nu = 1$ kHz.

### Performance analysis of 1 state MKF

In this section we discuss the performance of 1 and 2 state MKFs described in “Principle of 1 and 2 state multi-step Kalman filters” section. First we consider the 1 state MKF. For this, we set CFO to zero. Figure 3a shows the received constellation after CD and PMD compensation. The received symbols are processed by 1 state MKF to estimate and compensate phase noise. Figure 3b,c,d show the constellations of the received signal after 1 state MKF processing with $m=1, 10$ and 20 respectively. We observe that after MKF processing the constellations return to their original positions apart from the spread of symbols due to ASE noise.

Figure 4 shows the Q-factor curves as a function of launch powers for different step sizes of MKF. From Fig. 4 we can observe that for *m*= 10 and 20 , the Q-factor penalty is 0.68 dB and 1.32 dB at 1 dBm launch power when compared to the linear step KF. Since CFO is zero, the cycle slip in phase tracking is reduced and hence the Q factor is high even for large values of step-size *m*. This shows that phase can be estimated at the receiver using MKF with reduced sampling rate and a small penalty in the Q-factor value thus reducing the number of computations in comparison to the linear KF. For benchmark, we also compare the performance of 1 step MKF to the results of QPSK partitioning scheme^22^. We see that MKF outperforms the QPSK partitioning method. At lower launch powers, the QPSK partitioning scheme performance is closer to MKF; however, at higher launch powers, MKF performs significantly better over the QPSK partitioning algorithm even for step size as large as $m=20$. Thus, when the CFO is already estimated, say, as part of a multistage carrier recovery algorithm the residual time-varying phase noise can be tracked with reduced computational requirements by employing MKF with large step sizes.

Figure 5 shows the Q-factor variation with the number of spans to characterize long haul performance of the system. We calculated the Q-factor for various linewidths to determine the linewidth tolerance of the system under consideration. We found that for zero CFO, MKF shows a linewidth tolerance of 1 MHz before the performance deteriorates. Increasing the laser linewidth from 100 kHz to 1 MHz reduces Q-factor by $\approx$ 1.53 dB at launch power of 1 dBm. We see that performance of 1 state MKF is better than QPSK partitioning scheme over 40 spans.

### Performance analysis of 2 state MKF

Figure 6a shows the received constellation after CD and PMD compensation. This is then processed by the 2 state MKF for phase noise and CFO estimation and compensation. Figure 6b,c,d show the constellations of the received signal after 2 state MKF processing with $m = 1, 5$ and 10 respectively. We note that increasing the value of *m* increases the distortion in constellations . However, the constellation and the resulting BER is within the FEC limit for *m* as large as 10.

Figure 7 shows the Q-factor versus launch power curve for three different CFO values and for three different step sizes for $\delta \nu =100$ kHz. We see that for $m=1$, Q factor for CFO of 100 MHz and 1 GHz overlap each other indicating the suitability of 2 state MKF for estimation and tracking of CFO and phase noise. For $m=3$ and 5, and CFO of 100 MHz, the performance of the 2 state MKF reduces compared to $m=1$ case. However, the Q factor still remains significantly higher than the FEC limit as shown in Fig. 7. However at higher launch powers ($>2$ dBm) MKF with $m=5$ does not track CFO and phase noise as the deviation in the total actual phase and estimated phase increases due to CFO and NLPN.

Figure 8 shows the long haul performance of the 2 state MKF for 100 MHz at $m=1$ and 5 and for CFO = 1 GHz at $m=1$. At CFO as high as 1 GHz the proposed system can be operated upto 20 $\times$ 80 km. For CFO = 100 MHz the linear KF can go upto 30 spans but as we increase the step size the Q-factor values drops and the span over which data can be transmitted reduces. It can be attributed to the fact that the increase in step size decreases the symbol rate which in turn increases the overall phase rotation due to CFO.

Figure 9 shows the Q-factor values achieved for the various step sizes and various frequency offsets for 100 kHz laser linewidth. From the figure, we see that 2 state MKF with $m=1$ performs better than the other values of *m* for CFO in the range of 100 MHz to 1 GHz. For 100 MHz frequency offset step size upto 10 can be achieved with approximately 2.5 dB degradation in Q-factor value. We next varied the CFO from 100 MHz to 1.2 GHz. As seen from Fig. 9, for CFO $> 1$ GHz only $m=1$ can be achieved.

Figure 10 summarises the maximum step size *m* that can be achieved for the frequency offsets in the range 100 MHz to 1 GHz. For CFO $\le 1$ GHz, m is greater than 1, i.e. allowing the carrier recovery with reduced number of samples thereby increasing the computational efficiency. For 100 MHz CFO, step size up to $m=10$ can be achieved.

Figure 11 shows Q-factor versus launch power curve for 2 state MKF for three different values of m and $N_s=3$ spans. The results are compared with the block estimation based Kalman filter for carrier recovery^15^. For the system model proposed in Fig. 2 we simulated the block based Kalman filter for laser linewidth of 1 kHz and CFO of 100 MHz. Filter proposed in^15^ can track low laser linewidths and can reach the transmission distance of 320 km. On the contrary, the proposed 2 state Kalman filter can track laser linewidths upto 100 kHz over the transmission distance of 960 km.

### Dynamic frequency estimation

**Figure 12:**
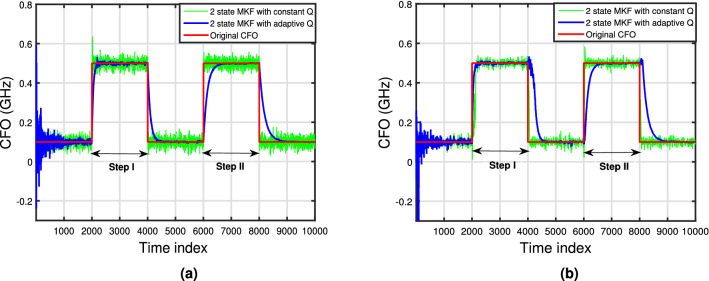
Tracked CFO by 2 state MKF with constant *Q* and adaptive *Q* for (**a**) m = 1 and (**b**) m = 3.

Figure 12 shows the tracking capability of the 2 state MKF with adaptive *Q* for the dynamic frequency offset case with $m=1$ and 3 compared with the 2 state MKF with constant *Q* over 12 span transmission link. The conventional MKF shows quick convergence but its accuracy is poor. On the other hand, the tracking capability of the MKF with adaptive *Q* is comparatively low but its estimation accuracy is better. This result is in agreement to the performance of the adaptive Kalman filter (AKF) proposed in^18^ for B2B system. Poor tracking capability of the filter can be attributed to the choice of $\beta$. A small value of $\beta$ reduces the convergence speed and the filter exhibits sluggish response in case of suddenly changing frequency offsets. On the other hand, larger values of $\beta$ gives rapid convergence but causes large dependence of *Q* on the innovation vector which can cause the filter to diverge. For $m=3$ system shows degradation in the tracking of frequency offset. This degradation can be reduced by detecting the symbols which are affected and then applying the cycle slip mitigation techniques^23^.

This dynamic behaviour in CFO is modeled as a step change in CFO by 0.5 GHz. We denote the step change in CFO during the initial part of transmission by step I and by step II, the step change in CFO during the later part of transmission. This allows us to bring out the effect of accumulated residual phase and frequency errors on the tracking ability for step change in CFO as described next. Exponential curve fit is done for step I and step II in Fig. 12 and rise time and fall times are computed. Rise time ($t_r$) is defined as number of symbols required to go from 100 MHz to 95% of 500 MHz and fall time ($t_f$) as 500 MHz to 95% of 100 MHz. Rise times and fall times for step $m= 1$ and 3 are tabulated in Table 1. From the values in Table 1, we can conclude that the tracking of CFO for step I is better than step II. This can be attributed to the fact that the rotation of constellation points at the step II symbol range is greater than step I symbol range thereby affecting the rise and fall of CFO with respect to the symbols hence affecting the convergence time. The proposed scheme shows the degradation in tracking of CFO for m = 3 compared to 1. But this degradation can be reduced by considering the final value of CFO after transition and then again applying the phase recovery scheme to all the symbols under transition.

**Table 1:** Rise times and fall times for step change in CFO.

Step no.	Step size (m = 1)		Step size (m = 3)	
	($t_r$)	($t_f$)	($t_r$)	($t_f$)
Step I	140	277	112	376
Step II	415	539	486	523

## Computational complexity of MKFs

In this section, we analyze the computational complexity of the proposed 1 state and 2 state MKFs in terms of total number of real additions, real multiplicaions and lookup tables (LUTs) required for implementation of the algorithm. Each real addition (RA) requires a real adder and real multiplication (RM) requires a real multiplier. We take RA, RM, and LUT as one unit of operation. The following rules govern the total number of real additions and real multiplications required for each operation.

1. Each matrix addition requires 4 RAs.
2. Each matrix inversion requires 6 RMs and 1 RA.
3. Each matrix multiplication requires 4 RAs and 8 RMs.
4. Each complex multiplication requires 3 RAs and 4 RMs.
5. The arg(), tan$^{-1}()$ operations requires a LUT.

The number of operations of 1 state and 2 state filters for each iteration are summarized in Table 2. From the entries in Table 2 and the rules given above we find that the 1 state MKF requires a total of 13 RAs, 12 RMs and 1 LUT. Similarly, the 2 state MKF requires a total of 66 RAs, 97 RMs and 2 LUTs.

The MKF reduces the computational complexity in comparison to the single-step Kalman filters by reducing the total number of computations by the factor of *m*:

11 $$\begin{aligned} \text {Reduction in no. of computations}=\text {Number of computations} \times \text {Total number of symbols} \times \left( 1-\frac{1}{m}\right) \end{aligned}$$

For example, the 2 state MKF with $m=5$ for 10,000 transmitted symbols, reduction in RMs$= 97 \times 10,000 \times \left( 1-\frac{1}{5}\right) =776000$ compared with single-step KF.

**Table 2:** Computational complexity for 1 state and 2 state MKF.

Operation	1 state MKF	2 state MKF
State prediction	1 RA	3 RA, 1 RM
Error covariance prediction	1 RA	2 MM, 1 MA
Kalman gain update	1 RA, 1 RM	1 MA, 3 MM, 1 MI
Error covariance update	1 RA, 2 RM	1 MA, 2MM
Measurement update	1 CM, 1 RM, 2 RA, 1 LUT	1 CM, 8 RM, 1 MA, 2 MM, 2 LUT
Averaging	1 RA	1 RA
CPE correction	1 CM	1 CM

RA, real addition; RM, real multiplication; MM, matrix Multiplication; MA, matrix addition; MI matrix inversion; CM complex multiplication; LUT lookup table.

## Conclusion

In this paper, we proposed 1 state and 2 state MKF for carrier tracking and estimation with reduced sampling rate requirements in 200 Gbps 16 QAM coherent transmission system and verified its performance with numerical simulations. Simulations are carried for long haul system (960 km) taking CD, PMD and nonlinearity in account. 1 state MKF is concerned with tracking for PN and NLPN. Simulation results show that MKF with *m* upto 20 can be used with $\le 1.5$ dB loss and results are found to perform better than QPSK partioning scheme. Also, maximum laser linewidth tolerance limit of the proposed 1 state MKF is found to be 1 MHz. 2 state MKF is concerned with joint mitigation of PN, NLPN, and CFO upto 1 GHz for $m \ge 1$. Proposed algorithm outperforms block based estimation using Kalman filter in terms of Q-factor values and number of spans transmission. Also, 2 state MKF was adapted in terms of *Q* so as to improve the estimation accuracy of CFO and results are found in agreement to the adaptive Kalman filter proposed in literature. Tracking performance for dynamic CFO was analysed for 2 state MKF, it was observed that 2 state MKF gives reduced jitter with adaptive *Q* in comparison to constant *Q*. Finally, we studied the computational complexity of the proposed 1 and 2 state MKF and computed the reduction in computations with slight degradation in performance. Our proposed filters use the linear Kalman filter resulting in significant computational advantage compared to nonlinear Kalman filters such as EKF and UKF thus making it suitable for joint mitigation of PN, NLPN, and CFO in high-rate high-modulation order coherent communication systems.

## References

[1] Ip E, Lau APT, Barros DJ, Kahn JM (2008). Coherent detection in optical fiber systems. Opt. Express.

[2] Li L (2017). A joint recovery scheme for carrier frequency offset and carrier phase noise using extended Kalman filter. Opt. Fiber Technol..

[3] Leven A, Kaneda N, Koc UV, Chen YK (2007). Frequency estimation in intradyne reception. IEEE Photon. Technol. Lett..

[4] Hoffmann S (2008). Frequency and phase estimation for coherent QPSK transmission with unlocked DFB lasers. IEEE Photon. Technol. Lett..

[5] Zhou X (2011). 64-Tb/s, 8 b/s/Hz, PDM-36QAM transmission over 320 km using both pre- and post-transmission digital signal processing. J. Lightw. Technol..

[6] Dris, S. et al. M-QAM carrier phase recovery using the viterbiviterbi monomial-based and maximum Likelihood Estimators. In *2013 Optical Fiber Communication Conference and Exposition and the National Fiber Optic Engineers Conference (OFC/NFOEC)*, 1–3 (IEEE, 2013).

[7] Wang Y, Serpedin E, Ciblat P (2003). Optimal blind carrier recovery for MPSK burst transmissions. IEEE Trans. Commun..

[8] Faruk MS, Savory SJ (2017). Digital signal processing for coherent transceivers employing multilevel formats. J. Lightwave Technol..

[9] Benani, A. M. & Gagnon, F. Comparison of carrier recovery techniques in M-QAM digital communication systems. In *2000 Canadian Conference on Electrical and Computer Engineering. Conference Proceedings*, 73–77. vol. 1 (2000).

[10] Barry JR, Kahn JM (1992). Carrier synchronization for homodyne and heterodyne detection of optical quadriphase-shift keying. J. Lightwave Technol..

[11] Jain A, Krishnamurthy PK (2016). Phase noise tracking and compensation in coherent optical systems using Kalman filter. IEEE Commun. Lett..

[12] Jain A, Krishnamurthy PK, Landais P, Anandarajah PM (2017). EKF for joint mitigation of phase noise, frequency offset and nonlinearity in 400 Gb/s PM-16-QAM and 200 Gb/s PM-QPSK systems. IEEE Photonics J..

[13] Jignesh J, Corcoran B, Lowery A (2016). Parallelized unscented Kalman filters for carrier recovery in coherent optical communication. Opt. Lett..

[14] Pfau, T. et al. Towards real-time implementation of coherent optical communication. *2009 Conference on Optical Fiber Communication*, 1–3 (2009).

[15] Inoue T, Namiki S (2014). Carrier recovery for M-QAM signals based on a block estimation process with Kalman filter. Opt. Express.

[16] Akhlaghi, S., Zhou, N. & Huang, Z. Adaptive adjustment of noise covariance in Kalman filter for dynamic state estimation. *2017 IEEE Power & Energy Society General Meeting*, 1–5 (2017).

[17] Seimetz M (2009). Transmitter design in high-order modulation for optical fiber transmission.

[18] Xiang Q, Yang Y, Zhang Q, Cao J, Yao Y (2019). Adaptive and joint frequency offset and carrier phase estimation based on Kalman filter for 16 QAM signals. Opt. Commun..

[19] Zhou X, Xie C (2016). Polarization and Nonlinear Impairments in Fiber Communication Systems in Enabling Technologies for High Spectral-Efficiency Coherent Optical Communication Networks.

[20] Zhang F (2011). Experimental comparison of different BER estimation methods for coherent optical QPSK transmission systems. IEEE Photonics Technol. Lett..

[21] Schmogrow R (2012). Error vector magnitude as a performance measure for advanced modulation formats. IEEE Photonics Technol. Lett..

[22] Fatadin I, Ives D, Savory SJ (2010). Laser linewidth tolerance for 16-QAM coherent optical systems using QPSK partitioning. IEEE Photonics Technol. Lett..

[23] Taylor MG (2009). Phase estimation methods for optical coherent detection using digital signal processing. J. Lightwave Technol..

[24] Serena, P., Bertolini, M. & Vannucci, A. Optilux toolbox. Available at optilux.sourceforge.net/Documentation/optilux-doc.pdf (2009).

